# How plants manage pathogen infection

**DOI:** 10.1038/s44319-023-00023-3

**Published:** 2023-12-19

**Authors:** Yinan Jian, Dianming Gong, Zhe Wang, Lijun Liu, Jingjing He, Xiaowei Han, Kenichi Tsuda

**Affiliations:** 1 National Key Laboratory of Agricultural Microbiology, Hubei Hongshan Laboratory, Hubei Key Laboratory of Plant Pathology, College of Plant Science and Technology, Huazhong Agricultural University, 430070 Wuhan, China; 2 Shenzhen Institute of Nutrition and Health, Huazhong Agricultural University, 430070 Wuhan, China; 3 Shenzhen Branch, Guangdong Laboratory for Lingnan Modern Agriculture, Genome Analysis Laboratory of the Ministry of Agriculture, Agricultural Genomics Institute at Shenzhen, Chinese Academy of Agricultural Sciences, 518120 Shenzhen, China; 4 National Key Laboratory of Crop Genetic Improvement, Hubei Hongshan Laboratory, Huazhong Agricultural University, 430070 Wuhan, China

**Keywords:** Plant Immunity, Pathogen Suppression, Antimicrobial Mechanism, Pathogen Virulence, Plant Microbiota, Immunology, Microbiology, Virology & Host Pathogen Interaction, Plant Biology

## Abstract

To combat microbial pathogens, plants have evolved specific immune responses that can be divided into three essential steps: microbial recognition by immune receptors, signal transduction within plant cells, and immune execution directly suppressing pathogens. During the past three decades, many plant immune receptors and signaling components and their mode of action have been revealed, markedly advancing our understanding of the first two steps. Activation of immune signaling results in physical and chemical actions that actually stop pathogen infection. Nevertheless, this third step of plant immunity is under explored. In addition to immune execution by plants, recent evidence suggests that the plant microbiota, which is considered an additional layer of the plant immune system, also plays a critical role in direct pathogen suppression. In this review, we summarize the current understanding of how plant immunity as well as microbiota control pathogen growth and behavior and highlight outstanding questions that need to be answered.

## Abstract (web summary)

Plants have evolved specific immune responses to directly suppress infiltrated pathogens. This review summarizes our current understanding of how plant immunity controls pathogen growth and behavior and highlights outstanding questions on pathogen suppression.

## Graphical abstract

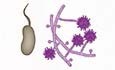

## Introduction

In nature, plants encounter diverse microbes including bacteria, fungi, oomycetes, and viruses. Some microbes are pathogens that impair plant growth and reproduction. Plants pre-form structural and molecular barriers and respond to infection by recognizing pathogen molecules through cell surface-localized pattern recognition receptors (PRRs) and intracellular nucleotide-binding domain leucine-rich repeat receptors (NLRs) (Ngou et al, 2022). PRRs recognize microbe-derived molecules (pathogen- or microbe-associated molecular patterns, PAMPs/MAMPs) as well as host-derived immunogenic molecules (damage-associated molecular patterns (DAMPs) and phytocytokines) to activate pattern-triggered immunity (PTI). NLRs recognize virulence proteins called effectors delivered by pathogens into the plant cell to activate effector-triggered immunity (ETI) (Ngou et al, 2022). Extracellular effectors can be recognized by PRRs, blurring the strict distinction of PTI and ETI (Lu & Tsuda, 2021). Activation of PTI and ETI results in various immune responses including oxidative burst (Mittler et al, 2022), calcium influx (Köster et al, 2022), activation of mitogen-activated protein kinase (MAPK) cascades (Sun & Zhang, 2022), transcriptional reprogramming (Tsuda & Somssich, 2015), phytohormone synthesis (Berens et al, 2017), callose deposition (Ellinger & Voigt, 2014), and programmed cell death (PCD) (Maekawa et al, 2023). Accumulating evidence suggests that PTI and ETI are intimately associated and trigger the overlapped immune responses (Lu & Tsuda, 2021; Yuan et al, 2021a; Ngou et al, 2021; Yuan et al, 2021b; Tian et al, 2021; Pruitt et al, 2021). From molecular genetic studies, it is well-established that these immune responses contribute to the suppression of pathogen growth. However, most of these immune responses do not directly explain how plant immunity stops pathogen infection. It has been also shown that plant-associated microbes contribute to direct pathogen suppression. How does plant immunity together with microbiota stop pathogen infection? In this review, we answer this question with a focus on bacterial, fungal, and oomycete pathogens and highlight important questions to stimulate future research.

## Physical barrier-mediated pathogen suppression

**Figure 1:**
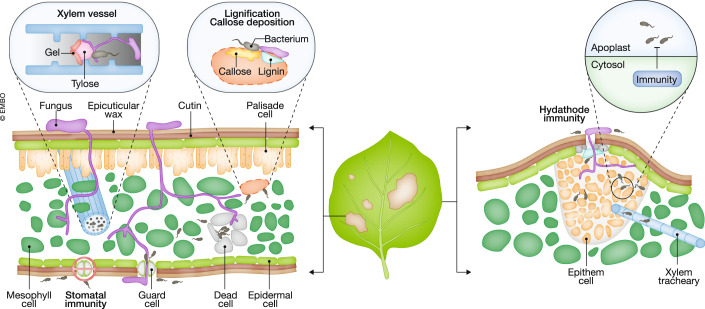
Physical barrier-mediated pathogen suppression. Plants suppress pathogen entry by closing stomata and the cuticle barrier. Once pathogens penetrate these physical barriers, plants remodel physical structures to suppress pathogen spread. Plants induce cell wall reinforcement including the deposition of callose and lignin. Xylem vessel is modified by tyloses and gels. Plant cells undergo localized cell death called hypersensitive response. These changes in physical structures contribute to the suppression of pathogen spread.

Plant tissues are nutrient-rich environments that attract pathogens. In order to access plant-derived nutrients, pathogens need to invade plant tissues through natural pores such as stomata and hydathodes or by penetrating physical structures. In turn, plants dynamically regulate these physical barriers to suppress pathogen invasion (Fig. 1).

### Pore immunity

Many foliar pathogens invade plants through stomata, pores in the epidermis of aerial tissues, which control gas exchange and water balance (Lin et al, 2022). Plants have evolved stomatal immunity that closes stomata upon pathogen infection via recognition of MAMPs, thereby restricting pathogen entry into leaf inner tissues (Melotto et al, 2006). In recent years, researchers have identified a variety of plant components and mechanisms in stomatal immunity, and we refer to recent reviews (Lin et al, 2022; Rodrigues & Shan, 2022). The importance of stomatal immunity is supported by the fact that pathogens have evolved to re-open stomata.

Some bacterial pathogens belonging to *Xanthomonas*, *Clavibacter*, and *Pseudomonas* invade leaf tissues via hydathodes, which are specialized glands at the leaf margin and directly connect to the xylem (Cerutti et al, 2019). Hydathodes are rich in water and nutrients that support microbial growth. In contrast to stomata, hydathodes do not close upon pathogen infection but do have post-invasive hydathode immunity which restricts the spread of non-adapted bacterial pathogens into the vasculature system (Paauw et al, 2023). While the required plant immune hubs BRI1-ASSOCIATED RECEPTOR KINASE 1 (BAK1) and ENHANCED DISEASE SUSCEPTIBILITY 1 - PHYTOALEXIN-DEFICIENT 4 - ACTIVATED DISEASE RESISTANCE 1 (EDS1-PAD4-ADR1) as well as pipecolic acid signaling have been identified, how hydathode immunity restricts pathogen spread remains unknown.

### Structural barriers

The plant cuticle is a lipid polymer layer with waxes present on the outermost surfaces of plant organs and serves as the front structural defense against pathogens. Plant cuticle is not static but is remodeled during pathogen invasion and is involved in plant immune signaling (Ziv et al, 2018). After penetrating the cuticle, pathogens encounter cell walls that are complex extracellular matrices composed of polysaccharides including cellulose, hemicellulose, and pectin (Dora et al, 2022). Many pathogens secrete cell wall-degrading enzymes to loosen cell walls for space and to access nutrients, but plants counter this by recognizing cell wall-degraded products as DAMPs to activate PTI (Dora et al, 2022). Modification and de novo synthesis of cell wall components occur during pathogen infection as an immune response. For instance, callose, which is de novo synthesized upon pathogen infection, is a part of papillae that appears to function as physical reinforcement to cell walls, thereby suppressing pathogen spread (Clay et al, 2009; German et al, 2023; Liu et al, 2023a; Wang et al, 2019a). Fungal cell walls contain β-1,3-glucan, the major polysaccharides of callose. Thus, β-1,3-glucan from callose may affect fungal behavior (Dora et al, 2022). Lignin and suberin are other types of cell wall reinforcement. Deposition of lignin and suberin is dynamically regulated and localized to the site of infection, thereby suppressing pathogen spread (Lee et al, 2019; Gallego-Giraldo et al, 2020; Kashyap et al, 2022; Fröschel et al, 2021). Plants may monitor the effectiveness of cell wall-mediated immunity as plants deficient in callose or lignin activate the phytohormone salicylic acid (SA)-mediated immunity (Gallego-Giraldo et al, 2011; Nishimura et al, 2003). In addition to cell wall reinforcement, tyloses and gels secreted by adjacent parenchyma cells form structural barriers in the xylem to restrict the spread of vascular bacterial pathogens (Leśniewska et al, 2017; Planas-Marquès et al, 2022).

### Programmed cell death

Plants kill their own cells (programmed cell death) to restrict pathogen spread and alert neighboring cells (Maekawa et al, 2023). While different forms of programmed cell death in animal immunity are well-defined, programmed cell death in plant immunity is defined as the hypersensitive response (HR) whose mechanism remains largely elusive. We refer to a recent review for the mechanism of programmed cell death in animal and plant immunity (Maekawa et al, 2023). HR is often induced during ETI and is considered to suppress pathogen spread and proliferation by acting as structural barrier and limiting pathogen growth space. Cellular components derived from dead cells can also activate immune responses by functioning DAMPs in neighboring cells. However, ETI activates various immune responses beyond HR. Thus, the causality of cell death for pathogen suppression remains elusive, largely due to the lack of genetic mutations that specifically block HR. Indeed, a type I metacaspase, AtMC1, is required to activate HR during ETI against the bacterial pathogen *Pseudomonas syringae* but not for resistance against the pathogen, decoupling HR from the suppression of pathogen growth (Coll et al, 2010). Consistent with this, ETI does not suppress the growth of ETI-triggering *P. syringae* strains in the infection site where HR occurs, while ETI strongly suppresses their population in the distal part of leaves where HR is not occurring (Jacob et al, 2023). Observed strong immune responses at the edge of the infection site further support this. Together, these results suggest that ETI associated with HR does not restrict the multiplication but spread of *P. syringae*. To what extent this holds true for other pathogens and plants needs to be explored.

## Molecule-mediated pathogen suppression

**Figure 2:**
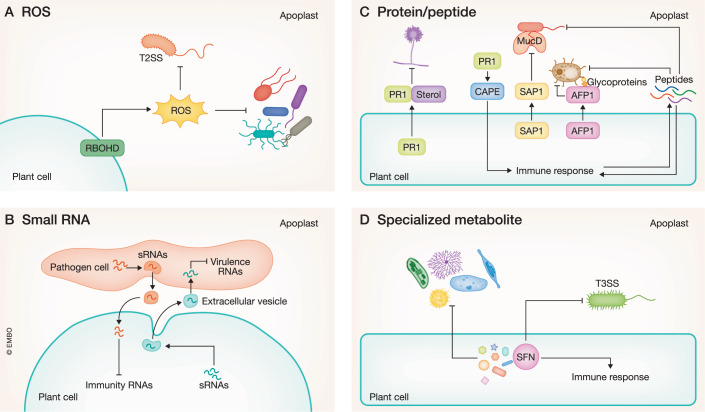
Molecule-mediated pathogen suppression. (**A**) Reactive oxygen species (ROS) directly suppresses pathogen growth by toxicity and virulence by dampening the type II secretion system (T2SS) of a potentially pathogenic *Xanthomonas*. (**B**) Plants secrete small RNAs (sRNAs) via extracellular vesicles (EVs) to suppress fungal virulence RNAs. *Botrytis cinerea* sends sRNAs via EVs to suppress plant immunity RNAs. (**C**) Pathogenesis-related protein 1 (PR1) sequesters sterol from the environment to suppress the sterol-auxotroph *Phytophthora*. The C-terminus of PR is cleaved to generate the CAPE peptide that activates plant immune responses. Secreted Aspartic Protease 1 (SAP1) cleaves a specific site in the *Pseudomonas syringae* protein MucD, thereby suppressing the pathogen. Antifungal protein 1 (AFP1) targets glycoproteins to suppress *Ustilago maydis*. Antimicrobial peptides have broad-spectrum activity and can also be specific to certain pathogens. (**D**) Specialized metabolites cause the loss of pathogen membrane integrity and also specifically target pathogen virulence. Sulforaphane (SFN) targets the type III secretion system (T3SS) of *Pseudomonas* and *Xanthomonas*.

Plants produce defense molecules that directly suppress pathogen growth and development in a constitutive and inducible manner. These molecules include reactive oxygen species (ROS), small RNAs, proteins/peptides, and specialized metabolites (Fig. 2).

### ROS

ROS plays a crucial role in diverse physiological processes of plants, in particular adaptive responses to biotic and abiotic stresses (Mittler et al, 2022). While the importance of ROS in plant immunity against a wide range of pathogens is well established, how ROS eventually leads to pathogen suppression is largely unknown. The importance of ROS in plant immunity is reflected by that pathogens scavenge ROS (Liu et al, 2019). ROS is generated in different cellular locations. For instance, the plasma membrane-localized NADPH oxidase RESPIRATORY BURST OXIDASE HOMOLOG D (RBOHD) produces the ROS O^2-^ in the extracellular space, which can then be readily converted to H_2_O_2_ via superoxide dismutase in the apoplast (Torres et al, 2002). Chloroplasts and mitochondria are also major locations of ROS production during plant immune responses (Mittler et al, 2022). ROS is sensed by a receptor and other redox-sensitive molecules to activate defense responses and regulates lignin deposition that ultimately leads to pathogen suppression (Dora et al, 2022). It is also widely believed that ROS directly inhibits pathogen growth because ROS as a reactive, toxic chemical can inhibit in vitro pathogen growth at high concentrations (Mittler et al, 2022; Zhang et al, 2023). However, direct evidence that ROS toxicity is the cause of pathogen suppression is scarce. Recently, it has been shown that RBOHD-mediated ROS directly suppresses the type II secretion system of a potentially harmful *Xanthomonas*, which makes the *Xanthomonas* non-pathogenic in *Arabidopsis thaliana* plants (Entila et al, 2023). This tamed *Xanthomonas* protected plants against the bacterial pathogen *P. syringae*, indicating that plant ROS acts as a signaling cue to modulate the behavior of microbes, turning a foe into a friend (Fig. 2A).

### Small RNAs

Small RNAs (sRNAs) are a group of short non-coding regulatory RNAs including microRNAs (miRNAs), small interfering RNAs (siRNAs), and transfer RNA fragments that play roles in diverse physiological processes (Zhan & Meyers, 2023). In addition to the role of sRNAs in the regulation of plant immune signaling, they also function in direct pathogen suppression (Fig. 2B). Cotton plants export the miRNAs miR159 and miR166 to target virulence genes encoding a cysteine protease and an isotrichidermin C-15 hydroxylase, respectively, of the fungal pathogen *Verticillium dahliae* (Zhang et al, 2016). *V. dahliae* expressing these genes resistant to miR159 and miR166 is hypervirulent, demonstrating the direct target of the plant miRNAs to the fungal virulence genes. *A. thaliana* plants also transfer sRNAs including miRNAs and siRNAs to the fungal pathogen *Botrytis cinerea* (Wang et al, 2016; Cai et al, 2018). Many transferred siRNAs target fungal genes that are involved in vesicle-trafficking pathways and are required for virulence (Cai et al, 2018; He et al, 2021). sRNA-mediated resistance appears widespread against filamentous pathogens. Secondary siRNAs derived from tetratricopeptide-repeat protein-encoding genes, whose production is mediated by miR161, target virulence and sporangia development genes of the oomycete pathogen *Phytophthora capsici* in *A. thaliana* plants (Hou et al, 2019). These plant sRNAs are cargos of extracellular vesicles (EVs) and the sRNA transfer to *B. cinerea* is mediated via EVs (Cai et al, 2018; Hou et al, 2019). Interestingly, *B. cinerea* also sends sRNAs into the plant cell via EVs to silence plant immunity genes (Weiberg et al, 2013; He et al, 2023). Whether EV-mediated sRNA transport from host plants to pathogens is a general plant defense strategy is still under debate (Zand Karimi et al, 2022), but these examples indicate that EVs are important carriers of sRNA weapons for both plants and pathogens. Whether sRNA-mediated direct pathogen suppression occurs for bacterial pathogens is unknown. However, artificial siRNAs expressed in transgenic *A. thaliana* plants can target virulence genes of *P. syringae* (Rastogi et al, 2019), suggesting that it is a possibility.

### Proteins/peptides

Pathogenesis-related (PR) proteins are traditionally named for plant proteins that are highly induced upon pathogen attack (Van Loon LC, 1985). Nowadays PR proteins are mostly pathogen-inducible, secreted, and potentially antimicrobial plant proteins (Van Loon et al, 2006). The role of the first discovered PR1 as an antimicrobial protein is based on two pieces of evidence: purified PR1 inhibits the growth of bacterial, fungal, and oomycete pathogens in vitro and *PR1* overexpression in plants suppresses pathogen infection (Alexander et al, 1993; Niderman et al, 1995; Rauscher et al, 1999). While the causal biochemical activity of PR1 for pathogen suppression remains largely elusive, genetic and biochemical evidence demonstrates that PR1 suppresses the sterol-auxotroph oomycete pathogen *Phytophthora* by sterol sequestration (Gamir et al, 2017). These results indicate that PR1 functions in direct pathogen suppression (Fig. 2C). However, the function of PR1 is also beyond its antimicrobial activity. The small peptide derived from the C-terminus of PR1 (CAPE1 in tomato and AtCAPE-PR1/CAPE9 in *A. thaliana*) is cleaved off by a cysteine protease into the CAPE peptide that functions as a phytocytokine, thereby activating plant immune responses (Chen et al, 2014; Chen et al, 2023). This makes the causal function of PR1 in pathogen suppression ambiguous (Fig. 2C). Nevertheless, the importance of PR1 in plant immunity is evident, reflected by the fact that pathogen effectors target PR1 (Breen et al, 2016; Li et al, 2022; Lin et al 2023). Other PR proteins such as PR2, PR5, defensin (PR12), and thionin (PR13) appear to have an activity to disrupt pathogen cell walls and the plasma membrane (Boccardo et al, 2019; Rayapuram et al, 2008; Zribi et al, 2021). The biochemical activity of these proteins leading to pathogen suppression in plants remains to be explored, but different PR proteins curiously form complexes to increase pathogen resistance (Han et al, 2023).

Other proteins apart from PR proteins also contribute to direct pathogen suppression. For instance, maize antifungal protein 1 (AFP1) belonging to secretory mannose-binding cysteine-rich receptor-like secreted protein family targets glycoproteins on the surface of the fungal pathogen *Ustilago maydis* to interfere with chitin metabolism, thereby blocking spore germination, cell budding, and growth (Ma et al, 2018; Ma et al, 2023) (Fig. 2C). Tomato leaf extract containing chitinases hydrolyzes hyphal tips of the fungus *Trichoderma viride*, which is inhibited by the chitin-binding Avr4 effector of the fungal pathogen *Cladosporium fulvum*, representing an example that chitinases can inhibit fungal growth (Mentlak et al, 2012). The oomycete pathogen *Phytophthora sojae* secretes the xyloglucan-specific endoglucanase PsXEG1 for virulence. Soybean plants secrete the glucanase inhibitor protein GmGIP1 that binds to PsXEG1 to block its virulence activity (Ma et al, 2017). Interestingly, the receptor of PsXEG1 RXEG1 encoding a PRR (LRR-RLP) from *Nicotiana benthamiana* also directly suppresses the activity of PsXEG1, thereby suppressing *P. sojae* virulence in addition to immune activation by RXEG1 (Sun et al, 2022). Secreted Aspartic Protease 1 (SAP1) of *A. thaliana* cleaves the *P. syringae* protein MucD at the specific site, which is required for virulence but not growth in vitro, thereby impairing pathogen colonization in *A. thaliana* plants (Wang et al, 2019b). *P*. *syringae* carrying MucD with a mutation at the cleavage site is hypervirulent and resistant to SAP1-mediated resistance. SAP1 is widely conserved in angiosperms and MucD in bacteria, pointing to the broad significance of SAP1-mediated antibacterial resistance. Interestingly, while *mucD* shows purifying selection overall, the SAP1 cleavage site is under positive selection among *Pseudomonas*, implying the arms race between plants and *Pseudomonas* via SAP1 and MucD (Fig. 2C).

Antimicrobial peptides (AMPs) are small proteins ranging from 15 to 150 amino acids (aa) with antibacterial, antifungal, and antioomycete activity and are ubiquitous in eukaryotes (Fig. 2C). AMPs are generally believed to have broad-spectrum activity, but more recent data show that they can be specific to certain microbes (Lazzaro et al, 2020). Due to their clinical values, a large number of AMPs from animal sources and de novo-designed AMPs have been described (Lazzaro et al, 2020). While research on plant-derived AMP-mediated pathogen suppression is largely lagging compared with the medical field, plant AMPs also function in suppressing pathogens in plants (Montesinos, 2023). For instance, MaSAMP (stable antimicrobial peptide) from *Microcitrus australasica* (Australian finger lime) inhibits infection by the vector-transmitted phloem-limited bacterial pathogen *Candidatus* Liberibacter asiaticus (*C*Las) which causes Citrus Huanglongbing disease, one of the most devastating and uncurable disease (Huang et al, 2021). While MaSAMP can kill *Liberibacter crescens*, a culturable *Liberibacter* strain, it also activates plant immune responses, suggesting its versatile role in plant immunity. Spray-applied MaSAMP is taken up by plants, stays for a week, and moves systemically through the vascular system where *C*Las colonizes, demonstrating its strong practical value in agriculture.

EVs released from tomato roots contain a wide variety of defense-related proteins and inhibit spore germination of fungal pathogens, such as *Fusarium oxysporum*, *B. cinerea*, and *Alternaria alternata* (De Palma et al, 2020). Interestingly, EVs from sunflower seedlings are taken up by the fungal pathogen *Sclerotinia sclerotiorum* and inhibit the spore germination and growth of the pathogen in vitro (Regente et al, 2017). These indicate that EV is a plant immune tool to deliver antimicrobial proteins/peptides to pathogens as for sRNAs.

### Specialized metabolites

Plants produce specialized metabolites that play a role in suppressing pathogens (Fig. 2D). Metabolites produced in a constitutive manner are called phytoanticipins and ones synthesized upon pathogen infection are called phytoalexins. The distinction between phytoanticipins and phytoalexins is ambiguous as the production of phytoanticipins can be increased or their activity can be regulated upon pathogen infection. Most specialized metabolites are specific to certain plant clades. We refer to recent reviews of detailed functions of specialized metabolites in plant immunity (Muñoz Hoyos & Stam, 2023; Kliebenstein, 2023). Many known defense-specialized metabolites such as glycoalkaloids, indoles, and terpenoids act at the microbial plasma membrane to cause the loss of membrane integrity, thereby suppressing pathogens (Muñoz Hoyos & Stam, 2023; Piasecka et al, 2015; Wang et al, 2023a).

Defense metabolites can target the fundamental microbial components leading to general growth suppression, but they also target specific virulence factors. For instance, sulforaphane (SFN), an isothiocyanate compound derived from aliphatic glucosinolates, inhibits *Pseudomonas* growth in vitro and non-host but not host-adapted *Pseudomonas* pathogens in *A. thaliana* plants (Fan et al, 2011). The *A. thaliana*-adapted *P. syringae* pathogens carry enzymes that metabolize SFN to overcome plant defense. SFN tolerance mechanism also exists in other bacterial pathogens belonging to *Xanthomonas* and *Pectobacterium* (Van den Bosch et al, 2020; Wang et al, 2023b), pointing to the significant role of SFN in plant immunity against bacterial pathogens. Interestingly, under concentrations that do not inhibit in vitro bacterial growth, SFN directly inhibits the central regulator of the type III secretion system (T3SS) of *P. syringae*, thereby suppressing pathogen virulence in *A. thaliana* plants (Wang et al, 2020). SFN also directly targets the virulence factor of *Xanthomonas* pathogens (Wang et al, 2022a). These results suggest that the major function of SFN might be to suppress bacterial virulence rather than general growth. Nevertheless, SFN also activates plant immune responses (Andersson et al, 2015; Schillheim et al, 2018). Thus, the role of SFN in plant immunity is versatile (Fig. 2D). Overall, the *in planta* mode of action of defense metabolites is poorly established, necessitating further research.

SA mediates plant immunity through activation of defense gene expression (Hou & Tsuda, 2022). In addition to this established role, SA is also implicated in direct pathogen suppression. SA can directly inhibit virulence gene expression of bacterial pathogens belonging to *Pseudomonas*, *Agrobacterium*, and *Pectobacterium* and endogenous SA concentrations in plants explain this phenomenon (Prithiviraj et al, 2005; Yuan et al, 2007; Wilson et al, 2017; Cooper, 2022). SA-deficient *A. thaliana* plants exhibit compromised suppression of the T3SS of *P. syringae* (Nobori et al, 2020). However, whether the direct role of SA in pathogen suppression is a major determinant and how SA directly suppresses pathogens remain to be investigated. When SA directly influences bacterial pathogens, it should localize in the apoplast where they colonize. SA also exists in the apoplast (Lim et al, 2020), but its cellular distribution remains unclear. Nevertheless, considering SA can influence the in vitro growth of certain bacteria isolated from *A. thaliana* roots and SA-degrading genes are common in plant-associated microbes, SA can be a key molecular signal that directly controls microbes (Lebeis et al, 2015; Nakano et al, 2022).

## Nutrient-, water-, and pH-mediated pathogen suppression

**Figure 3:**
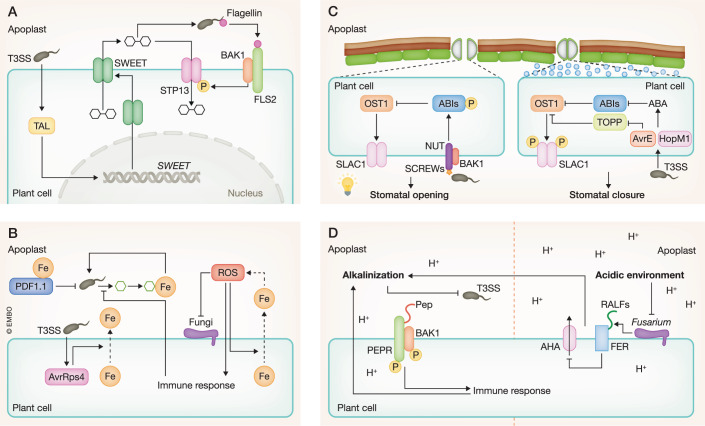
Nutrient-, water-, and pH-mediated pathogen suppression. (**A**) Sugar-mediated suppression. TAL effectors directly activate the expression of sugar transporter SWEET genes, increasing sugar contents in the apoplast. The pattern recognition receptor (PRR) complex FLS2-BAK1 activates the sugar transporter STP13 via phosphorylation to sequester sugars from the apoplast. (**B**) Iron-mediated suppression. A secreted plant defensin PDF1.1 sequesters iron to reduce iron contents in the apoplast to suppress *Pectobacterium carotovorum*. Plant immunity targets the iron acquisition system of *Pseudomonas syringae*. A type III effector AvrRps4 promotes iron accumulation in the apoplast. Iron accumulates at the infection site of *Blumeria graminis* f. sp. *tritici*, associated with reactive oxygen species (ROS) accumulation which promotes iron efflux. This iron-ROS positive feedback contributes to pathogen resistance. (**C**) Water-mediated suppression. The type III effectors AvrE and HopM1 activate ABA accumulation and signaling to trigger stomatal closure, causing aqueous environments. The phytocytokines SCREWs activate the PRR complex NUT-BAK1 to activate the negative regulators of ABA signaling (ABIs) to promote stomatal opening. Constant light activates SA signaling to promote stomatal opening. These prohibit pathogens from creating aqueous environments. (**D**) pH-mediated suppression. PTI triggers apoplast alkalinization that inhibits the expression of the bacterial type III secretion system (T3SS) and promotes the Damage-Associated Molecular Pattern (DAMP) Pep-mediated immunity through the pH sensor in the Pep receptor PEPR. The fungal pathogen *Fusarium oxysporum* secretes a functional homolog of the plant regulatory peptide RALF to induce alkalinization, thereby causing plant disease.

In addition to specific immune-related molecules described above, common molecules such as sugars, water, and protons also directly affect pathogen growth and virulence (Fig. 3).

### Nutritional immunity

Pathogens actively increase nutrient availability. For instance, the effector PsAvh413 from *P. sojae* interacts with and enhances the enzymatic activity of soybean trehalose-6-phosphate synthase 6 to increase trehalose accumulation in soybean, thereby increasing a carbon source of the pathogen and promoting infection (Zhu et al, 2023). The bacterial pathogen *Xanthomonas oryzae* introduces transcription-activator-like (TAL) effectors to directly activate SWEET sugar transporter genes to increase apoplastic sugars, which benefits pathogen growth (Chen et al, 2010) (Fig. 3A). The fungal pathogen *Rhizoctonia solani* also induces expression of a SWEET gene (Gao et al, 2018). The sugar transporter STP8 appears to be translocated from the endoplasmic reticulum to the host-derived extrahaustorial membrane in *A. thaliana* plants upon infection with the fungal pathogen *Golovinomyces cichoracearum*, which increases sugar availability to the pathogen (Liu et al, 2021). In turn, plants activate the expression of a sugar transport protein gene encoding STP13 that mediates sugar uptake from the apoplast to deprive sugars in the apoplast, thereby suppressing *B. cinerea* infection (Lemonnier et al, 2014). STP13 is also posttranslationally activated via phosphorylation by the PRR complex FLS2 and BAK1 upon infection with *P. syringae* (Yamada et al, 2016) (Fig. 3A). This leads to reduced sugar concentrations and restricted bacterial proliferation in the apoplast. Whether reduced sugar affects pathogen growth due to limited nutrition remains uncertain as reduced sugar concentration is also associated with reduced T3SS activity (Yamada et al, 2016). Plants appear to sequester various nitrogen and carbon sources from the leaf apoplast as a part of immune responses as commensal and avirulent *P. syringae* show nutrient starvation response but virulent *P. syringae* counteracts this (Nobori et al, 2022). Consistently, the plant urea transporter *AtDUR3*, which sequesters urea from the apoplast (Bohner et al, 2015), is induced by infection with commensal and avirulent *P. syringae* but suppressed with virulent *P. syringae* (Nobori et al, 2022).

Iron is an essential nutrient for plants as well as pathogens. It is thought that pathogens acquire iron from the host and the host may reduce iron availability as a resistance mechanism. Consistent with this, a *P. syringae* type III effector, AvrRps4, increases iron accumulation in the apoplast by targeting the plant iron sensor protein BRUTUS to facilitate iron uptake and proliferation of *P. syringae* in *A. thaliana* plants (Xing et al, 2021) (Fig. 3B). Pathogen infection often triggers iron deficiency response in plants, and iron deficiency activates plant immune responses mediated by phytohormones including SA and ethylene (Segond et al, 2009; Shen et al, 2016; Trapet et al, 2021; Platre et al, 2022). Notably, *A. thaliana* plants secrete an iron-chelating defensin protein to activate iron deficiency-mediated immune response via ethylene signaling, thereby limiting infection with the necrotrophic bacterial pathogen *Pectobacterium carotovorum* (Hsiao et al, 2017) (Fig. 3B). Nevertheless, whether plants actively sequester iron from the apoplast in order to reduce iron availability to pathogens remains elusive. Apart from being an essential nutrient, iron can also function as a defense chemical. H_2_O_2_ can undergo the Fenton reaction, which generates extremely reactive hydroxy radicals in the presence of excess free Fe^2+^. Although whether the Fenton reaction occurs in plants is unknown, wheat plants increase local iron concentrations associated with the increase of ROS in the apoplast. In turn, ROS activates cytosolic iron depletion and iron efflux in plants. This positive feedback loop contributes to the suppression of the fungal pathogen *Blumeria graminis* f. sp. *tritici* (Liu et al, 2007) (Fig. 3B). *In planta* bacterial transcriptome under plant immune activation identifies the iron acquisition pathway of *P. syringae* as the major target of plant immunity (Nobori et al, 2018). Both PTI and ETI shut down the expression of the bacterial iron acquisition pathway, as bacteria themselves do under iron-rich conditions, without affecting iron concentration in the apoplast, suggesting that plants interfere with the iron pathway by generating a false signal to the bacteria (Fig. 3B).

Constitutive activation of a MAPK, MPK6, leads to the suppression of T3SS. This associates with reduced levels of aspartic acid, citric acid, and 4-hydroxybenzoic acid that induce the expression of *P. syringae* T3SS. Supplementation of those metabolites restores T3SS expression and virulence of *P. syringae* (Anderson et al, 2014). Whether plant immunity actively restricts those metabolites remains to be investigated.

### Water immunity

Pathogens create an aqueous habitat in the apoplast of infected leaves by introducing virulence effectors, thereby causing water-soaking, a typical symptom of plant disease, especially under high humidity (Aung et al, 2018). This indicates that pathogens benefit from water-rich environments. For instance, *P. syringae* introduces type III effectors, AvrE and HopM1, to activate the phytohormone abscisic acid (ABA) biosynthesis and signaling. This triggers stomatal closure leading to water-soaking that supports pathogen multiplication (Xin et al, 2016; Hu et al, 2022; Roussin-Léveillée et al, 2022) (Fig. 3C). Interestingly, AvrE is a water-permeable channel, suggesting that *P. syringae* also directly increases water availability by its own channel inserted into the plant plasma membrane (Nomura et al, 2023). An important question is whether plants restrict water to suppress pathogens. Earlier studies showing that *P. syringae* experiences water stress during ETI suggest that plants restrict water (Wright & Beattie, 2004; Freeman & Beattie, 2009). Indeed, plants secrete and sense phytocytokines, SMALL PHYTOCYTOKINES REGULATING DEFENSE AND WATER LOSS (SCREWs) via the cognate receptor PLANT SCREW UNRESPONSIVE RECEPTOR (NUT) to inhibit ABA signaling and stomatal closure. This promotes stomatal opening and consequently apoplastic water loss, leading to the disruption of aqueous habitats, thereby suppressing *P. syringae* growth (Liu et al, 2022a) (Fig. 3C). More recently, it has been shown that constant light increases SA accumulation which likely counteracts ABA-mediated stomatal closure, thereby promoting stomatal opening. This prevents *P. syringae* from creating aqueous environments to suppress *P. syringae* growth (Lajeunesse et al, 2023) (Fig. 3C).

### pH change-mediated immunity

Plants dynamically change apoplastic pH during development and stress via various mechanisms, mainly through H^+^-ATPases and anion channels (Geilfus, 2017). It has been long known that apoplastic alkalization is a part of PTI (Bolwell et al, 1995) (Fig. 3D). Although the mechanism and consequence of apoplastic alkalization are not entirely clear, there is a clue that increased pH directly suppresses pathogen infection. For instance, T3SS of bacterial pathogens are pH sensitive (Van Dijk et al, 1999). The increased pH in the apoplast would decrease the secretion of effectors mediated by T3SS to suppress bacterial virulence (Fig. 3D). In addition to the direct effect on pathogens, apoplastic alkalization is sensed by cell-surface immune receptors (plant elicitor peptides to its receptors; PEPRs) through the pH sensor Glu/Asp to support PEPR function, thereby promoting plant immunity (Liu et al, 2022b) (Fig. 3D).

Plant growth-promoting rhizobacteria *Pseudomonas capeferrum* WCS358 secretes organic acids to lower pH, thereby suppressing plant immune responses (Yu et al, 2019). *F. oxysporum* secretes a functional homolog of the plant regulatory peptide RALF (rapid alkalinization factor) to induce alkalinization, thereby causing disease in plants (Masachis et al, 2016) (Fig. 3D). Thus, the consequence of apoplastic pH changes depends on pathogens, and further research is required to investigate the effects of pH on diverse pathogens.

## Microbiota-mediated pathogen suppression

**Figure 4:**
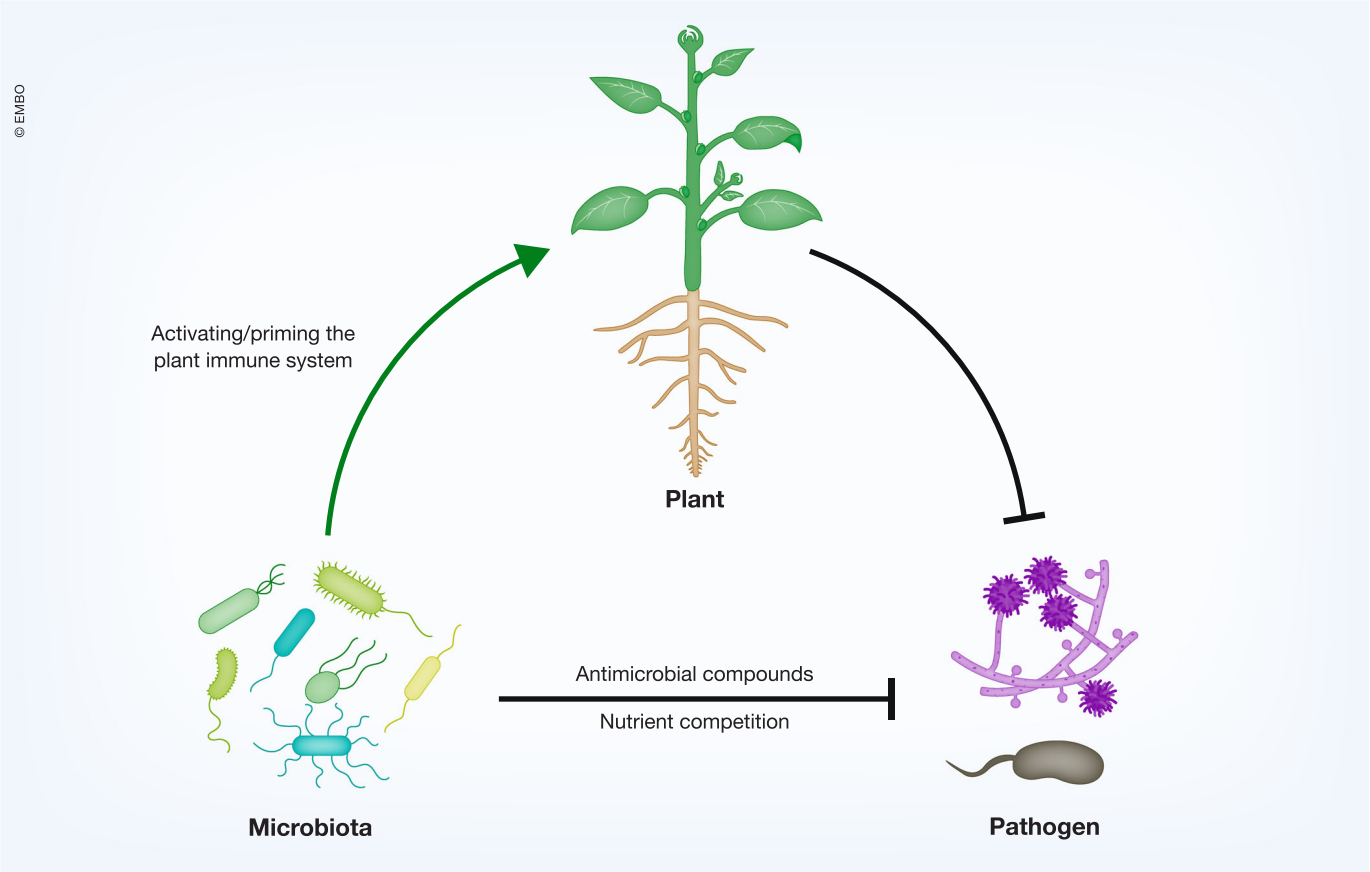
Microbiota-mediated pathogen suppression. Plant microbiota directly suppresses pathogen infection by producing antimicrobial compounds and nutrient competition and indirectly through the activation of plant immunity.

Plants are associated with diverse microbes called the plant microbiota that collectively contributes to plant health (Fig. 4). Accumulating evidence suggests that the plant innate immune system controls the structure and function of the plant microbiota which is required for gating proper plant immune status and serves as the additional layer of the plant innate immune system (Ma et al, 2021; Chen et al, 2020; Durán et al, 2018; Paasch et al, 2023). So-called disease-suppressive soils have the ability to protect plants against pathogens (Carrión et al, 2019). Plants also actively recruit beneficial microbes upon pathogen infection (Berendsen et al, 2018; Liu et al, 2023b). Numerous studies have shown that plant-associated microbes support resistance against pathogens indirectly through the activation of plant immunity (Pereira et al, 2023; Paasch et al, 2023). A recent study has shown that phyllosphere microbiota increases plant production of branched-chain amino acids such as leucine that suppress the fungal pathogen *Ustilaginoidea virens* by inducing apoptosis-like cell death via H_2_O_2_ overproduction in the pathogen (Liu et al, 2023c). We do not further discuss microbiota-mediated immune activation as it comes to the point of how plant immunity stops pathogens. Below we discuss examples of direct pathogen suppression by microbiota (Box 1).

In need of answers

- Are different antimicrobial mechanisms responsible for immunity activated by different receptors?
- To what extent do antimicrobial mechanisms show the specificity to different pathogens?
- What is the relative contribution of pathogen suppression by plant microbiota?
- How can we utilize the knowledge of direct pathogen suppression in agricultural practice?

### Space and nutrient competition

Microbiota members and pathogens share common resources such as space and nutrients, therefore they compete in the common niche (Caballero-Flores et al, 2023; Pereira et al, 2023). The idea of space competition is simple. When someone (microbiota) is sitting on the chair, you (pathogen) cannot sit on the same chair. While the concept of space competition is appreciated by the plant microbiota community and demonstrated in animal microbiota fields (Caballero-Flores et al, 2023), evidence supporting the notion that plant microbiota suppresses pathogen infection by space competition is rather scarce. On the other hand, there is accumulating evidence for microbiota-mediated pathogen suppression via nutrient competition. For instance, iron is an essential element for microbes, but iron bioavailability in the environment can be limited as it is predominantly present as insoluble ferric (Fe^3+^) oxide which is not readily available for microbes (Kramer et al, 2020). As plants do (Liang, 2022), microbes produce iron-chelating molecules called siderophores which help microbes acquire iron from the environment (Kramer et al, 2020). A siderophore can be specific to the producer and other microbes cannot use it. Alternatively, it can be a common good and other microbes can take it up together with iron. Gu et al found that rhizosphere microbiota competes for iron to suppress the bacterial pathogen *Ralstonia solanacearum*, thereby protecting tomato plants (Gu et al, 2020). Notably, rhizosphere bacteria secreting pathogen-inhibitory siderophores suppress the pathogen in vitro and in soil environments, while rhizosphere bacteria secreting pathogen-promotive siderophores facilitate pathogen infection. This suggests that certain microbiota bacteria produce siderophores which the pathogen cannot use to sequester iron from the environment causing iron limitation to the pathogen, thereby suppressing pathogen growth. Similarly, *A. thaliana*-protecting commensal *Pseudomonas* against pathogenic *Pseudomonas* is attributed to siderophore-mediated iron acquisition (Shalev et al, 2022). The beneficial rhizobacterium *Bacillus velezensis* SQR9 utilizes the type VII secretion system to secrete YukE which is inserted into the plant plasma membrane and causes iron leakage from the host plant (Liu et al, 2023d). While increased iron availability promotes root colonization by SQR9, which benefits plants on the one hand, it can also attract pathogens on the other hand. Together, this indicates that microbiota members can suppress or facilitate pathogen infection via iron, depending on the condition, and points to the complex regulation of iron in nature. Further research is required to figure out how to control plant disease by using microbiota members that influence iron availability.

### Antimicrobial molecules

Biocontrol by plant-protective microbes against pathogens attracted huge attention from researchers and farmers. Numerous studies have shown that certain microbes can directly suppress pathogen infection by producing antimicrobial metabolites. Here, we discuss recent examples showing direct pathogen suppression by antimicrobial molecules of native microbiota members. *Pseudomonas piscium* from wheat head microbiota secrets phenazine-1-carboxamide to directly affect the fungal protein FgGcn5, which results in misregulation of histone acetylation in *Fusarium graminearum*, thereby suppressing pathogen growth and virulence (Chen et al, 2018). Interestingly, *Pantoea agglomerans* from fungal fruiting bodies of *F. graminearum* in rice plants produce herbicolin A that is responsible for the suppression of *F. graminearum* (Xu et al, 2022). Thus, bacterial microbiota members associated with a pathogen also suppress the growth of the pathogen although its physiological significance is unknown. *Pseudomonas mosselii* isolated from rice rhizosphere produces pseudoiodinine that directly suppresses the growth of *Xanthomonas* pathogens and the fungal pathogen *Magnaporthe oryzae* in vitro and in rice plants (Yang et al, 2023). The rice seed bacterial endophyte *Sphingomonas melonis* produces anthranilic acid that interferes with the sigma factor RpoS of the seed-borne bacterial pathogen *Burkholderia plantarii*, thereby suppressing pathogen virulence and growth (Matsumoto et al, 2021). Interestingly, *S. melonis* is accumulated and transmitted across generations in seeds of resistant but not susceptible rice plants of the same genotype, indicating that disease resistance is determined by the seed endophyte but not the rice genotype and resistance can be provided to susceptible rice genotypes by the endophyte accumulation. Myxobacteria inhibits the growth of the oomycete pathogen *P. sojae* by producing a thiaminase that scavenges thiamine required for *P. sojae* growth (Xia et al, 2023). The thiaminase is secreted via outer membrane vesicles, pointing to the significant role of EVs in microbe-microbe interactions that determine plant-pathogen interactions.

### Other mechanisms

Plant protection against *P. syringae* by *Rhizobium* Leaf202 isolated from healthy *A. thaliana* leaves requires the type VI secretion system of the *rhizobium* (Vogel et al, 2021). The plant-associated beneficial bacterium *Pseudomonas putida* IsoF uses the type IVB secretion system to protect tomato plants against *R. solanacearum* (Purtschert-Montenegro et al, 2022). These results suggest that some microbiota-mediated pathogen suppression requires direct cell-cell contact as these bacterial secretion systems directly inject toxic effectors into the competitor cell.

Modification of pH changes by microbiota also contributes to pathogen suppression. The rhizobacterium *Rahnella aquatilis* secretes gluconic acid, which results in the acidification of rhizosphere that counteracts *F. oxysporum*-induced alkalization, thereby suppressing the pathogen in tomato plants (Palmieri et al, 2020). Curiously, soil acidification influences bacterial communities and reduces the resistance of peanuts against *Fusarium* pathogens. Microbiota from acidified soils have a reduced ability to prevent *Fusarium* infection (Li et al, 2023). Thus, soil acidification can have the opposite effect on *Fusarium* infection, and further research is warranted to understand the effect of pH on disease resistance in natural settings.

## Perspectives

Important questions in plant immunity are do plants eliminate pathogens and if so, is pathogen elimination adaptive for plants? There are mutualistic microbes that obviously provide fitness advantages to plants. For instance, Arbuscular mycorrhiza fungi associate with most land plants to provide phosphate to the host plant. Nitrogen-fixing and nodule-forming rhizobia benefit legume plants by providing nitrogen (Wang et al, 2022b). In these cases, microbial colonization is adaptive and plants have specific receptors to identify their friends. However, in nature, plants do not only associate with these mutualists but also other microbes that include commensal and pathogens. Thus, immune responses triggered by other microbes can suppress these mutualists, which would decrease plant fitness. In addition, synthetic microbiota reconstitution experiments using microbial strains isolated from healthy plants indicate that plants require bacterial colonization for survival in the presence of fungi and oomycetes (Durán et al, 2018). Thus, at least bacteria are essential components for plants to survive in natural environments but they also attack plants. How do plants deal with this dilemma? One possibility is that plant immunity has sufficient selectivity toward distinct microbes. While there is a certain level of selectivity at the microbial recognition by immune receptors (Colaianni et al, 2021; Parys et al, 2021), this selectivity is not sufficient for all the cases as pathogens and mutualists can have identical MAMPs that would trigger the same immune responses. Then, do plant immune outputs such as the production of defense protein and metabolites that directly regulate microbial growth and behavior have sufficient selectivity (Box 1)? Several studies have shown that immune outputs have selectivity. For instance, the very common immune output ROS burst affects only certain bacterial colonization. ROS specifically dampens the activity of a potentially pathogenic *Xanthomonas* and turns it into plant protective bacterium (Entila et al, 2023). Together with other studies (Wang et al, 2019b; Nobori et al, 2018; Wang et al, 2020), this suggests that a major microbial target of plant immunity is the virulence mechanism. Once pathogen virulence is shut down, microbes become commensal, leading to healthy co-habitation of plants and microbes. Thus, plant immunity does not simply suppress microbes but changes microbial behavior to promote plant fitness. To what degree this holds true needs further investigation. To this end, more research is needed to better understand how plant immunity controls diverse microbes in natural settings.

Plant immune responses are activated by different receptors. Do these receptors activate the same antimicrobial mechanisms? For instance, it has been shown that early transcriptional responses to seven different MAMPs, DAMPs, and phytocytokines, which are recognized by different receptors, are mostly overlapped with quantitative differences (Bjornson et al, 2021). Moreover, transcriptional reprogramming in PTI and ETI is largely overlapped with temporal differences (Mine et al, 2018). Do these quantitative differences result in distinct antimicrobial mechanisms? To answer these questions, we need to characterize antimicrobial mechanisms responsible for pathogen suppression in various situations (Box 1).

Plant immune activation is often associated with growth defects and yield penalties (He et al, 2022). Thus, enhancing plant disease resistance by strengthening plant immunity or activating plant immune responses by immune elicitors is challenging due to the growth-defense tradeoff. Direct pathogen suppression does not involve activation of plant immune responses leading to the tradeoff. Therefore, engineering plants with enhanced mechanisms of direct pathogen suppression has the potential to provide increased disease resistance while mitigating the growth-defense tradeoff to maintain crop yield (Box 1).
